# Neurons as Canonical Correlation Analyzers

**DOI:** 10.3389/fncom.2020.00055

**Published:** 2020-06-30

**Authors:** Cengiz Pehlevan, Xinyuan Zhao, Anirvan M. Sengupta, Dmitri Chklovskii

**Affiliations:** ^1^John A. Paulson School of Engineering and Applied Sciences and Center for Brain Science, Harvard University, Cambridge, MA, United States; ^2^Center for Neural Science, New York University, New York, NY, United States; ^3^Rutgers, The State University of New Jersey, New Brunswick, NJ, United States; ^4^Center for Computational Biology, Flatiron Institute, New York, NY, United States; ^5^Langone Medical Center, New York University, New York, NY, United States

**Keywords:** neural networks, Canonical Correlation Analysis (CCA), Hebbian plasticity, pyramidal neuron, biologically plausible learning

## Abstract

Normative models of neural computation offer simplified yet lucid mathematical descriptions of murky biological phenomena. Previously, online Principal Component Analysis (PCA) was used to model a network of single-compartment neurons accounting for weighted summation of upstream neural activity in the soma and Hebbian/anti-Hebbian synaptic learning rules. However, synaptic plasticity in biological neurons often depends on the integration of synaptic currents over a dendritic compartment rather than total current in the soma. Motivated by this observation, we model a pyramidal neuronal network using online Canonical Correlation Analysis (CCA). Given two related datasets represented by distal and proximal dendritic inputs, CCA projects them onto the subspace which maximizes the correlation between their projections. First, adopting a normative approach and starting from a single-channel CCA objective function, we derive an online gradient-based optimization algorithm whose steps can be interpreted as the operation of a pyramidal neuron. To model networks of pyramidal neurons, we introduce a novel multi-channel CCA objective function, and derive from it an online gradient-based optimization algorithm whose steps can be interpreted as the operation of a pyramidal neuron network including its architecture, dynamics, and synaptic learning rules. Next, we model a neuron with more than two dendritic compartments by deriving its operation from a known objective function for multi-view CCA. Finally, we confirm the functionality of our networks via numerical simulations. Overall, our work presents a simplified but informative abstraction of learning in a pyramidal neuron network, and demonstrates how such networks can integrate multiple sources of inputs.

## 1. Introduction

As neural networks evolved for competitive behaviorally-relevant tasks, it is natural to model them using a normative approach, where one starts from a principled objective function and derives an online optimization algorithm that models an operation of a neural system. Such approach often leads to simplified and interpretable models for complex biological phenomena. Perhaps, the most famous example of this is modeling a neuron as an online PCA algorithm (Oja, 1982). This model accounts for the weighted summation of synaptic inputs in a single, linear, point (single-compartment) neuron and Hebbian synaptic plasticity (synaptic weight is proportional to the correlation of pre- and post-synaptic activity). Recently, Oja's model of a single neuron has been extended to a network of neurons by deriving it from a multi-channel-PCA objective function (Pehlevan et al., 2015). In addition to the phenomena explained by the Oja model, the extension (Pehlevan et al., 2015) accounted for the anti-Hebbian learning rules of the lateral synaptic connections (synaptic weight is proportional to the negative of the correlation between pre- and post-synaptic activity). Because of their analytical tractability, the output of such network models can be predicted for any input.

However, there is mounting experimental evidence that a point neuron is an extreme oversimplification. Many neurons contain multiple segregated dendritic compartments which integrate synaptic inputs separately and can each have a membrane potential different from that of the soma (Poirazi and Mel, 2001; Polsky et al., 2004). Neuronal firing often requires coincident input onto distal and proximal dendrites (Larkum et al., 2009; Larkum, 2013). Moreover, synaptic plasticity is the function of the neuronal output (spiking) and membrane potential in the corresponding compartment (Bittner et al., 2015).

Multiple influential, biophysically grounded models have been proposed to describe the computational role of dendritic compartmentalization and nonlinearities (Poirazi and Mel, 2001; Poirazi et al., 2003; Polsky et al., 2004; Jadi et al., 2014; Urbanczik and Senn, 2014; Alemi et al., 2017; Guerguiev et al., 2017; Haga and Fukai, 2017). These models provide mechanistic and computational descriptions of the active membrane processes that account for experimental observations.

In this paper, we start from computational principles first and apply a normative approach to networks of multi-compartment neurons to derive algorithmic descriptions of their function. Specifically, we propose to model information processing in pyramidal neurons as online CCA algorithms (Hotelling, 1992; Yang et al., 2019). Because we derive these models from principled objective functions, we can predict the output of the network for any input analytically.

CCA is a natural choice for extending the normative modeling of point-neuron networks as PCA algorithms (Oja, 1982, 1992; Földiak, 1989; Rubner and Tavan, 1989; Sanger, 1989; Pehlevan et al., 2015) to multi-compartment neuron networks. In such neurons different dendritic compartments receive inputs from different sources, for example, top-down and bottom-up inputs, or inputs from multiple modalities. Information from these multiple sources can be integrated using CCA by linearly projecting each of the datasets onto low dimensional subspaces with maximal cross-correlation (Parise and Ernst, 2016). For Gaussian data, CCA can be optimal for various objectives such as, for example, mutual information (Chechik et al., 2005), but its utility extends to real-world applications as well (Painsky and Tishby, 2017).

Previous work on neural network implementations of CCA include (Lai and Fyfe, 1999; Pezeshki et al., 2003; Vía et al., 2007; Haga and Fukai, 2017), all of which use point neurons and non-local learning rules. In biology, synaptic learning rules must be local i.e., depend only on the information available in the corresponding pre- and post-synaptic neurons. In Lai and Fyfe (1999), there are two or three output neurons, and the synapses that innervate one of them need to have access to the activity of another neuron to update their strength. In Pezeshki et al. (2003), an estimate for the covariance matrix of the data needs to be stored and be globally available to all neurons. In Vía et al. (2007), update rules again involve numerous nonlocal computations.

We make the following contributions:

1. We interpret an existing online algorithm for single-channel CCA, as the operation of a two-compartment pyramidal neuron.
2. We derive a pyramidal neuron network from a novel objective function for multi-channel CCA.
3. We derive a model of a neuron with more than two dendritic compartments from an objective function for a single-channel multi-view CCA.

The rest of the paper is organized as follows. In section 2, we derive an online algorithm from the single-channel CCA objective function and map it onto the operation of a pyramidal neuron. In section 3, we explain why the standard objective function for multi-channel CCA yields a biologically implausible network of pyramidal neurons. In section 4, we propose a novel objective function for multi-channel CCA from which we derive a biologically plausible network of pyramidal neurons. In section 5, we derive a model of a neuron with more than two dendritic compartments from a single-channel multi-view CCA objective function. In section 6, we provide numerical simulation results for these neuronal algorithms.

## 2. A Pyramidal Neuron as an Online Single-Channel CCA Algorithm

Given two datasets with the same number of data points but in different subspaces, a single-channel CCA projects them onto a common line so that the two unit-variance projections are maximally correlated. Let us represent each pair of data points as ${\text{x}}_{t}\in {\mathbb{R}}^{n}$ and ${\text{y}}_{t}\in {\mathbb{R}}^{m}$, *t* = 1, …, *T*. The CCA objective function has the following form:

(1) $$\begin{array}{l} \underset{a\in {\mathbb{R}}^{n},b\in {\mathbb{R}}^{m}}{\max}\;\frac{1}{T}\sum_{t=1}^{T}\left(a^{⊤}x_{t}\right)\left(b^{⊤}y_{t}\right),\\ \text{ s}.\text{t}.\;\frac{1}{T}\sum_{t=1}^{T}{\left(a^{⊤}x_{t}\right)}^{2}=1,\frac{1}{T}\sum_{t=1}^{T}{\left(b^{⊤}y_{t}\right)}^{2}=1. \end{array}$$

In Supplementary Information A, we provide CCA's analytical solutions and their various properties for completeness.

We would like to rewrite (1) in a form amenable to a biologically plausible online algorithm. One way of doing so is by completing a square with constant terms (see constraints):

(2) $$\begin{array}{l} \text{arg}\underset{\text{a},\text{b}}{\text{max}}\frac{1}{T}\overset{T}{\underset{t=1}{\sum^{\;}}}\left({\text{a}}^{⊤}{\text{x}}_{t}\right)\left({\text{b}}^{⊤}{\text{y}}_{t}\right)\\ =\text{arg}\underset{\text{a},\text{b}}{\text{max}}\frac{1}{T}\overset{T}{\underset{t=1}{\sum^{\;}}}\left[\left({\text{a}}^{⊤}{\text{x}}_{t}\right)\left({\text{b}}^{⊤}{\text{y}}_{t}\right)+\frac{1}{2}{\left({\text{a}}^{⊤}{\text{x}}_{t}\right)}^{2}+\frac{1}{2}{\left({\text{b}}^{⊤}{\text{y}}_{t}\right)}^{2}\right]\\ =\text{arg}\underset{\text{a},\text{b}}{\text{max}}\frac{1}{2T}\overset{T}{\underset{t=1}{\sum^{\text{​}}}}{\left({\text{a}}^{⊤}{\text{x}}_{t}+{\text{b}}^{⊤}{\text{y}}_{t}\right)}^{2}\\ \text{ s.t. }\frac{1}{T}\overset{T}{\underset{t=1}{\sum^{\;}}}{\left({\text{a}}^{⊤}{\text{x}}_{t}\right)}^{2}=1,\frac{1}{T}\overset{T}{\underset{t=1}{\sum^{\;}}}{\left({\text{b}}^{⊤}{\text{y}}_{t}\right)}^{2}=1. \end{array}$$

Next, we introduce the constraints into the objective using the method of Lagrange multipliers and regroup the terms:

(3) $$\begin{array}{l} \underset{\text{a},\text{b}}{\max}\underset{\alpha ,\beta }{\;\min}\frac{1}{2T}\sum_{t=1}^{T}{\left(a^{⊤}x_{t}+b^{⊤}y_{t}\right)}^{2}-\frac{\alpha }{2}\left(\frac{1}{T}\sum_{t=1}^{T}{\left(a^{⊤}x_{t}\right)}^{2}-1\right)\\ -\frac{\beta }{2}\left(\frac{1}{T}\sum_{t=1}^{T}{\left(b^{⊤}y_{t}\right)}^{2}-1\right)\\ \underset{a,b}{\max}\underset{\alpha ,\beta }{\text{ min}}\frac{1}{2T}\sum_{t=1}^{T}[{\left(a^{⊤}x_{t}+b^{⊤}y_{t}\right)}^{2}-\alpha \left({\left(a^{⊤}x_{t}\right)}^{2}-1\right)\\ -\frac{\beta }{2}\left({\left(b^{⊤}y_{t}\right)}^{2}-1\right)]. \end{array}$$

To simplify the notation below we define the following variables:

(4) $$\begin{array}{l} c_{t}^{a}:={\text{a}}^{⊤}{\text{x}}_{t},\;c_{t}^{b}:={\text{b}}^{⊤}{\text{y}}_{t},\;c_{t}:=c_{t}^{a}+c_{t}^{b}. \end{array}$$

We solve (3) using stochastic gradient ascent/descent with respect to **a**, **b**, α, and β.

(5) $$\begin{array}{l} {\text{a}}_{t+1}={\text{a}}_{t}+\eta_{a}\left(c_{t}{\text{x}}_{t}-\alpha_{t}c_{t}^{a}{\text{x}}_{t}\right),\;{\text{b}}_{t+1}={\text{b}}_{t}+\eta_{b}\left(c_{t}{\text{y}}_{t}-\beta_{t}c_{t}^{b}{\text{y}}_{t}\right),\\ \alpha_{t+1}=\alpha_{t}+\frac{\eta_{\alpha }}{2}\left({\left(c_{t}^{a}\right)}^{2}-1\right),\;\beta_{t+1}=\beta_{t}+\frac{\eta_{\beta }}{2}\left({\left(c_{t}^{b}\right)}^{2}-1\right). \end{array}$$

**Figure 1:**
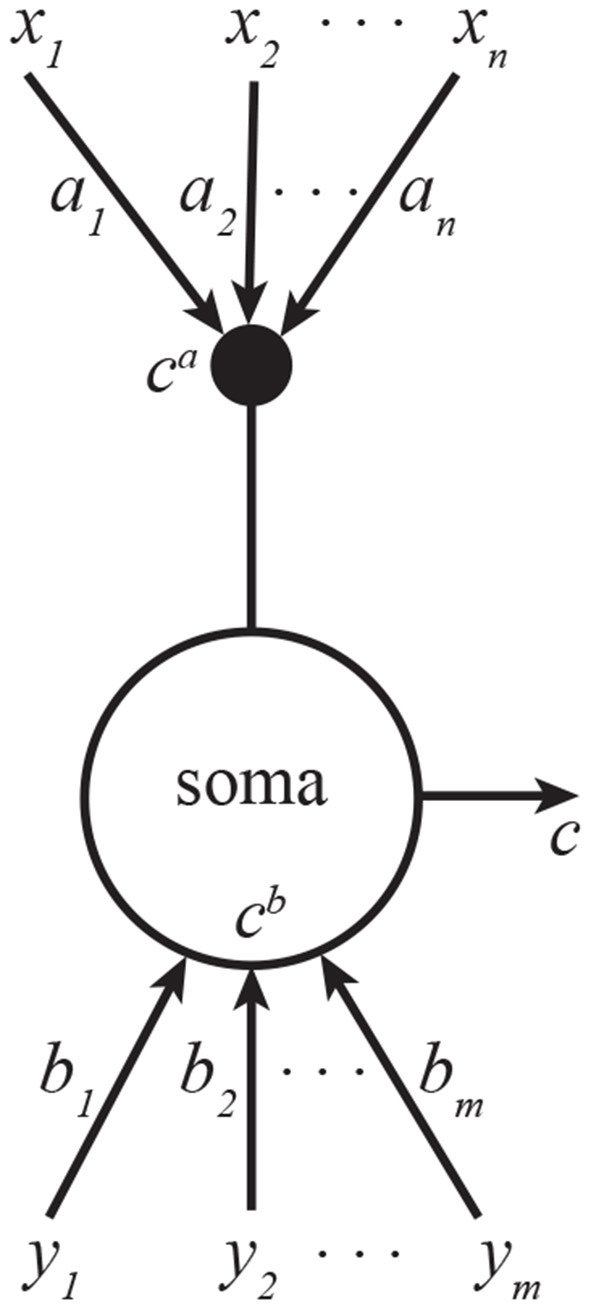
A CCA model of a pyramidal neuron. One set of inputs innervate proximal dendrites, another—distal. The neuron outputs the sum of projections onto a common line. Subscripts in this figure denote indices of vector elements.

We can interpret this algorithm as the operation of a pyramidal neuron. Here, $c_{t}^{b}$ and $c_{t}^{a}$ are proximal and distal dendritic currents, *c*_*t*_ is the output of the pyramidal neuron, **b**_*t*_ is a vector of proximal synaptic weights, **a**_*t*_ is a vector of distal synaptic weights, and α_*t*_ and β_*t*_ are scalar variables confined to proximal and distal dendrites respectively, and ηs are learning rates (Figure 1). The fixed point of this Algorithm corresponds to the solution of Equation (1) (see Supplementary Information B).

To see how these learning rules can be implemented biologically, we note that they can be expressed solely in terms of total output, *c*, signaled by backpropagating spikes (Stuart et al., 1997) and the distal dendritic current, *c*^*a*^, signaled by the calcium plateau potentials. The key to this is the relationship *c* = *c*^*a*^ + *c*^*b*^ in Equation (4) which allows expressing the learning rules only in terms of the two available signals. Remarkably, such non-Hebbian plasticity rules have been reported experimentally (Golding et al., 2002; Bittner et al., 2017; Magee and Grienberger, 2020).

Although algorithm (5) can be found in Lai and Fyfe (1999) (up to a redefinition of variables α_*t*_ and β_*t*_) it was interpreted there as the learning dynamics of a network with two point neurons and non-local learning rules. Our interpretation of the algorithm as operation of a pyramidal neuron will allow us to generalize it to multi-channel CCA implemented by a network of neurons.

## 3. The Standard Multi-Channel CCA Requires Biologically Implausible Interactions

Given two datasets with the same number of data points but in different vector spaces, a multi-channel CCA projects them onto a common vector space so that the different components of the same projection are uncorrelated but the corresponding components of the two projections are maximally correlated. Given the success of our approach in the previous section, one may want to derive a network of neurons from the standard multi-channel CCA cost function:

(6) $$\begin{array}{l} \underset{\begin{array}{c} {\text{a}}_{1},\ldots ,{\text{a}}_{d},\\ {\text{b}}_{1},\ldots ,{\text{b}}_{d} \end{array}}{\max}\frac{1}{T}\overset{d}{\underset{i=1}{\sum }}\overset{T}{\underset{t=1}{\sum }}\left({\text{a}}_{i}^{⊤}{\text{x}}_{t}\right)\left({\text{b}}_{i}^{⊤}{\text{y}}_{t}\right),\\ \text{s}.\text{t}.\frac{1}{T}\overset{T}{\underset{t=1}{\sum }}\left({\text{a}}_{i}^{⊤}{\text{x}}_{t}\right)\left({\text{a}}_{j}^{⊤}{\text{x}}_{t}\right)=\delta_{ij},\\ \;\frac{1}{T}\overset{T}{\underset{t=1}{\sum }}\left({\text{b}}_{i}^{⊤}{\text{y}}_{t}\right)\left({\text{b}}_{j}^{⊤}{\text{y}}_{t}\right)=\delta_{ij},\;i,j=1,\ldots ,d. \end{array}$$

Following the same derivation procedure as in section 2, we complete the squares and use the method of Lagrange multipliers to arrive at:

(7) $$\begin{array}{l} \underset{\begin{array}{c} a_{1},\ldots ,a_{d},\\ b_{1},\ldots ,b_{d} \end{array}}{\max}\underset{\begin{array}{c} \alpha_{1},\ldots \alpha_{d},\\ \beta_{1},\cdots ,\beta_{d} \end{array}}{\min}\underset{\begin{array}{c} A_{ij},B_{ij},\\ i,j=1,\ldots ,d,i\neq j \end{array}}{\min}\frac{1}{2T}\sum_{i=1}^{d}\sum_{t=1}^{T}[{\left(a_{i}^{⊤}x_{t}+b_{i}^{⊤}y_{t}\right)}^{2}\\ -\alpha_{i}\left({\left(a_{i}^{⊤}x_{t}\right)}^{2}-1\right)-\beta_{i}\left({\left(b_{i}^{⊤}y_{t}\right)}^{2}-1\right)]\\ -\frac{1}{2T}\sum_{i=1}^{d}\sum_{\begin{array}{c} j=1,\\ j\neq i \end{array}}^{d}A_{ij}\sum_{t=1}^{T}\left(a_{i}^{⊤}x_{t}\right)\left(a_{j}^{⊤}x_{t}\right)\\ -\frac{1}{T}\sum_{i=1}^{d}\sum_{\begin{array}{c} j=1,\\ j\neq i \end{array}}^{d}B_{ij}\sum_{t=1}^{T}\left(b_{i}^{⊤}y_{t}\right)\left(b_{j}^{⊤}y_{t}\right). \end{array}$$

The first two lines of (7) has *d* copies of the Lagrangian formulation for a single-channel CCA (3) suggesting that its online optimization can correspond to the operation of *d* pyramidal neurons. However, the interactions between these neurons given by *d*(*d* − 1) constraints in the third and fourth lines of (7) lead to a biologically implausible algorithm (see Supplementary Information C). Indeed, the decorrelation of proximal (as well as distal) currents among different neurons requires neurons to communicate information about such currents to each other. Yet, biological neurons do not output proximal or distal currents separately, only their sum.

To solve this problem, in the next section, we present a new objective function for CCA where the constraints are formulated in terms of neural outputs.

## 4. A Network of Pyramidal Neurons Derived From a Novel Multi-Channel CCA Objective

To derive a biologically plausible multi-channel CCA algorithm we resort to deflation: assuming we know the top *d* − 1 canonical variable pairs we find the *d*th canonical variable pair. We formulate a CCA objective with constraints expressed in terms of neural outputs accessible to other neurons based on the following proposition.

**Proposition 1**. *Given the top d* − 1 *canonical variable pairs*, **a**_1_, …, **a**_*d*−1_ *and* **b**_1_, …, **b**_*d*−1_, *the solution to the following optimization problem gives the dth pair of canonical variables*:

(8) $$\begin{array}{l} \underset{\text{a}\in {\mathbb{R}}^{n},\text{b}\in {\mathbb{R}}^{m}}{\max}\frac{1}{T}\overset{T}{\underset{t=1}{\sum }}\left({\text{a}}^{⊤}{\text{x}}_{t}\right)\left({\text{b}}^{⊤}{\text{y}}_{t}\right),\\ \text{s}.\text{t}.\frac{1}{T}\overset{T}{\underset{t=1}{\sum }}{\left({\text{a}}^{⊤}{\text{x}}_{t}\right)}^{2}=1,\frac{1}{T}\overset{T}{\underset{t=1}{\sum }}{\left({\text{b}}^{⊤}{\text{y}}_{t}\right)}^{2}=1,\\ \frac{1}{T}\overset{T}{\underset{t=1}{\sum }}\left({\text{a}}^{⊤}{\text{x}}_{t}+{\text{b}}^{⊤}{\text{y}}_{t}\right)\left({\text{a}}_{i}^{⊤}{\text{x}}_{t}+{\text{b}}_{i}^{⊤}{\text{y}}_{t}\right)=0,\;i=1,\ldots ,d-1. \end{array}$$

*Proof:* See Supplementary Information D.     □

Starting from the optimization problem (8) and following the steps that lead to (5), one can derive an online algorithm for the *d*th canonical variable pair. The optimization problem in the Lagrange multiplier formulation has the following form:

(9) $$\begin{array}{l} \underset{a,b}{\max}\underset{\alpha ,\beta ,m_{i}}{\min}\frac{1}{T}\sum_{t=1}^{T}[\frac{1}{2}{\left(a^{⊤}x_{t}+b^{⊤}y_{t}\right)}^{2}-\frac{\alpha }{2}\left({\left(a^{⊤}x_{t}\right)}^{2}-1\right)\\ -\frac{\beta }{2}\left({\left(b^{⊤}y_{t}\right)}^{2}-1\right)-\sum_{i=1}^{d-1}m_{i}\left(a_{i}^{⊤}x_{t}+b_{i}^{⊤}y_{t}\right)\left(a^{⊤}x_{t}+b^{⊤}y_{t}\right)] \end{array}$$

We define the following variables

(10) $$\begin{array}{l} c_{d,t}^{a}:={\text{a}}_{t}^{⊤}{\text{x}}_{t},\;c_{d,t}^{b}:={\text{b}}_{t}^{⊤}{\text{y}}_{t},\\ c_{i,t}:={\text{a}}_{i,t}^{⊤}{\text{x}}_{t}+{\text{b}}_{i,t}^{⊤}{\text{y}}_{t},\;i=1,\ldots ,d-1,\\ c_{d,t}:=c_{d,t}^{a}+c_{d,t}^{b}-\overset{d-1}{\underset{i=1}{\sum }}m_{i,t}c_{i,t}, \end{array}$$

and optimize using stochastic gradient/ascent:

(11) $$\begin{array}{l} {\text{a}}_{d,t+1}={\text{a}}_{d,t}+\eta_{a}\left(c_{d,t}{\text{x}}_{t}-\alpha_{d,t}c_{d,t}^{a}{\text{x}}_{t}\right),\\ {\text{b}}_{d,t+1}={\text{b}}_{d,t}+\eta_{b}\left(c_{d,t}{\text{y}}_{t}-\beta_{d,t}c_{d,t}^{b}{\text{y}}_{t}\right),\\ m_{i,t+1}=m_{i,t}+\eta_{m}\left(c_{d,t}^{a}+c_{d,t}^{b}\right)c_{i,t},\;i=1,\ldots ,d-1,\\ \alpha_{d,t+1}=\alpha_{d,t}+\frac{\eta_{\alpha }}{2}\left({\left(c_{d,t}^{a}\right)}^{2}-1\right),\\ \beta_{t+1}=\beta_{t}+\frac{\eta_{\beta }}{2}\left({\left(c_{d,t}^{b}\right)}^{2}-1\right). \end{array}$$

In addition, we replace $c_{d,t}^{a}+c_{d,t}^{b}$ with *c*_*d,t*_ to make the algorithm biologically plausible. Our modification to learning rule for *m*_*i*_ is:

(12) $$\begin{array}{l} \;m_{i,t+1}=m_{i,t}+\eta_{m}\left(c_{d,t}^{a}+c_{d,t}^{b}\right)c_{i,t}\\ \text{changes to}\;m_{i,t+1}=m_{i,t}+\eta_{m}c_{d,t}c_{i,t}. \end{array}$$

To see why the modified rule works, assume that the weight updates converged to a stationary state, i.e. $0=\langle \Delta m_{i}\rangle =\langle \left(c_{d}^{a}+c_{d}^{b}\right)c_{i}\rangle -m_{i}$, where we used 〈*c*_*i*_*c*_*j*_〉 = δ_*ij*_ (Supplementary Information A) and the brackets denote an average over the inputs. The desired CCA solution for the *d*^th^ canonical variable pair, i.e., *m*_*i*_ = 0 and $\langle \left(c_{d}^{a}+c_{d}^{b}\right)c_{i}\rangle =0$ satisfies this condition. Even though other solutions exist, in simulations (section 6), we see that the algorithm converges to only the desired one.

The resulting algorithm can be implemented by pyramidal neurons. As before, we interpret $c_{d,t}^{a}$, $c_{d,t}^{b}$, and *c*_*d,t*_ as distal, proximal, and total output currents, respectively, **a**_*d,t*_, **b**_*d,t*_, as distal and proximal synaptic weight vectors, α_*d,t*_, β_*d,t*_ as distal and proximal dendritic variables. We interpret *c*_*i,t*_ as the outputs of neurons that were trained to extract *i*th (*i* < *d*) pair of canonical variables and *m*_*di*_ are lateral weights from those neurons. Note that these lateral weight updates are anti-Hebbian.

The above derivation assumed that all *i* = 1, ⋯ , *d* − 1 canonical components are already extracted successfully, each by a different pyramidal neuron. To compute CCA from scratch, one way is to extract the *d* components sequentially: *i*th neuron has to wait for the result of (*i* − 1)th neuron. Once the distal and proximal weights of the (*i* − 1)th neuron converges, they are frozen and one moves to the next neuron. A subtle point here is about the lateral connections. Algorithm defined by (10) and (11) omits lateral connections between previous neurons in the sequence, suggesting that lateral connections should be removed once a neuron's weights converges. Such removal naturally happens because lateral weights decay to zero at the fixed point of the algorithm.

**Figure 2:**
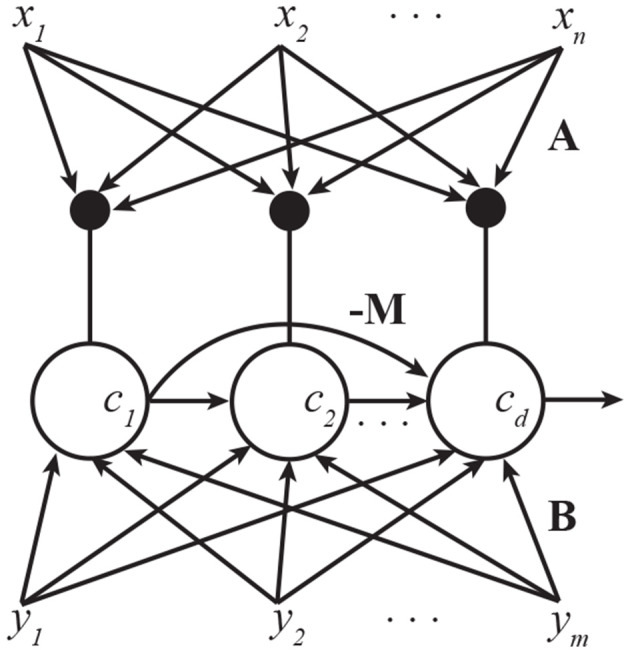
A pyramidal neuron network that implements multi-channel CCA. Lateral connections are anti-Hebbian and asymmetric. Subscripts in this figure denote indices of vector elements.

However, it is also possible, and more biologically plausible, to train all pyramidal neurons simultaneously using an asymmetric network architecture (Figure 2). This is akin to the asymmetric lateral connectivity in the Generalized Hebbian Network (Sanger, 1989) and APEX (Diamantaras and Kung, 1996) network for PCA. The resulting algorithm is given in Algorithm 1.

**Algorithm 1:** CCA network

**Input:** Parameters *d*, η_*a*_, η_*b*_, η_α_, η_β_ and η_*m*_. Initial α_1_, …, α_*d*_ and β_1_, …, β_*d*_. Initial synaptic weights **A** ∈ ℝ^*n*×*d*^, **B** ∈ ℝ^*m*×*d*^, and **M** ∈ ℝ^*d*×*d*^. *M*_*ij*_ = 0 for *j* ≥ *i*. **for** *t* = 1, 2, 3, …, *T* **do** // Neural activity Receive inputs **x**_*t*_ and **y**_*t*_ **for** *i* = 1, …, *d* **do** Calculate proximal and distal dendritic currents: $c_{i,t}^{a}=\overset{n}{\underset{j=1}{\sum }}A_{ji,t}x_{j,t}$, $c_{j,t}^{a}=\overset{m}{\underset{j=1}{\sum }}B_{ji,t}y_{j,t}$, Calculate pyramidal neuron outputs: $c_{i,t}=c_{i,t}^{a}+c_{i,t}^{b}-\overset{i-1}{\underset{j=1}{\sum }}M_{ij,t}c_{j,t}$ **end for** // Synaptic and homeostatic plasticity Update synaptic weights: $A_{ij,t+1}=A_{ij,t}+\eta_{a}\left(c_{j,t}x_{i,t}-\alpha_{j,t}c_{j,t}^{a}x_{i,t}\right)$, *i* = 1, …, *n, j* = 1, …, *d* $B_{ij,t+1}=B_{ij,t}+\eta_{b}\left(c_{j,t}y_{i,t}-\beta_{j,t}c_{j,t}^{b}y_{i,t}\right)$, *i* = 1, …, *m, j* = 1, …, *d* *M*_*ij,t*+1_ = *M*_*ij,t*_ + η_*m*_*c*_*i,t*_*c*_*j,t*_, *i* = 1, …, *d*, *j* < *i* Update dendritic variables: $\alpha_{i,t+1}=\alpha_{i,t}+\frac{\eta_{\alpha }}{2}\left({\left(c_{i,t}^{a}\right)}^{2}-1\right)$,*i* = 1, …, *d* $\beta_{i,t+1}=\beta_{i,t}+\frac{\eta_{\beta }}{2}\left({\left(c_{i,t}^{b}\right)}^{2}-1\right)$,*i* = 1, …, *d* **end for**

## 5. Neurons With Multiple Dendritic Branches as Multiview Canonical Correlation Analyzers

In this section, we return to the model of a single pyramidal neuron and extend it to neurons with more than two dendritic compartments using a multiview single-channel CCA, i.e., CCA on multiple sets of variables (Kettenring, 1971). There are multiple ways to generalize CCA to multiple sets of variables; the version we are using here is “SUMCOR” (sum of correlations): given a dataset with *k* views, $\{{\text{x}}_{t}^{\left(i\right)}\in {\mathbb{R}}^{n(i)}$, *t* = 1, …, *T*, *i* = 1, …, *k*}, the SUMCOR version of multiview CCA projects each dataset on the common line that maximizes the sum of pairwise correlations between them. Formally, we consider the following SUMCOR objective function (Kettenring, 1971):

(13) $$\begin{array}{l} \underset{{\text{a}}^{\left(1\right)},{\text{a}}^{\left(2\right)},\cdots \;,{\text{a}}^{\left(k\right)}}{\max}\frac{1}{T}\underset{i<j}{\sum }\overset{T}{\underset{t=1}{\sum }}\left({{\text{a}}^{\left(i\right)}}^{⊤}{\text{x}}_{t}^{\left(i\right)}\right)\left({{\text{a}}^{\left(j\right)}}^{⊤}{\text{x}}_{t}^{\left(j\right)}\right),\;\\ \text{ s}.\text{t}.\frac{1}{T}\overset{T}{\underset{t=1}{\sum }}{\left({{\text{a}}^{\left(i\right)}}^{⊤}{\text{x}}_{t}^{\left(i\right)}\right)}^{2}=1,\;i=1,2,\cdots \;,k \end{array}$$

From now on, the SUMCOR version of multiview CCA is simply referred to as multiview CCA.

In order to derive a neural algorithm for multiview CCA, as before we complete the square in the objective and introduce Lagrange multipliers:

(14) $$\begin{array}{l} \underset{{\text{a}}^{\left(1\right)},{\text{a}}^{\left(2\right)},\cdots \;,{\text{a}}^{\left(k\right)}}{\max}\underset{\alpha^{\left(1\right)},\alpha^{\left(2\right)},\cdots \;,\alpha^{\left(k\right)}}{\min}\frac{1}{2T}\overset{T}{\underset{t=1}{\sum }}{\left(\overset{k}{\underset{i=1}{\sum }}{{\text{a}}^{\left(i\right)}}^{⊤}{\text{x}}_{t}^{\left(i\right)}\right)}^{2}\\ -\overset{k}{\underset{i=1}{\sum }}\frac{\alpha^{\left(i\right)}}{2}\left(\frac{1}{T}\overset{T}{\underset{t=1}{\sum }}{\left({{\text{a}}^{\left(i\right)}}^{⊤}{\text{x}}_{t}^{\left(i\right)}\right)}^{2}-1\right) \end{array}$$

Defining

(15) $$\begin{array}{l} c_{t}^{\left(i\right)}:={{\text{a}}_{t}^{\left(i\right)}}^{⊤}{\text{x}}_{t}^{\left(i\right)},\;c_{t}:=\overset{k}{\underset{i=1}{\sum }}c_{t}^{\left(i\right)} \end{array}$$

We optimize this objective by gradient descent/ascent:

(16) $$\begin{array}{l} {\text{a}}_{t+1}^{\left(i\right)}={\text{a}}_{t}^{\left(i\right)}+\eta_{a}^{\left(i\right)}\left(c_{t}-\alpha_{t}^{\left(i\right)}c_{t}^{\left(i\right)}\right){\text{x}}_{t}^{\left(i\right)},\;\alpha_{t+1}^{\left(i\right)}=\alpha_{t}^{\left(i\right)}+\frac{\eta_{\alpha }^{\left(i\right)}}{2}\left(c_{t}^{\left(i\right)2}-1\right) \end{array}$$

**Figure 3:**
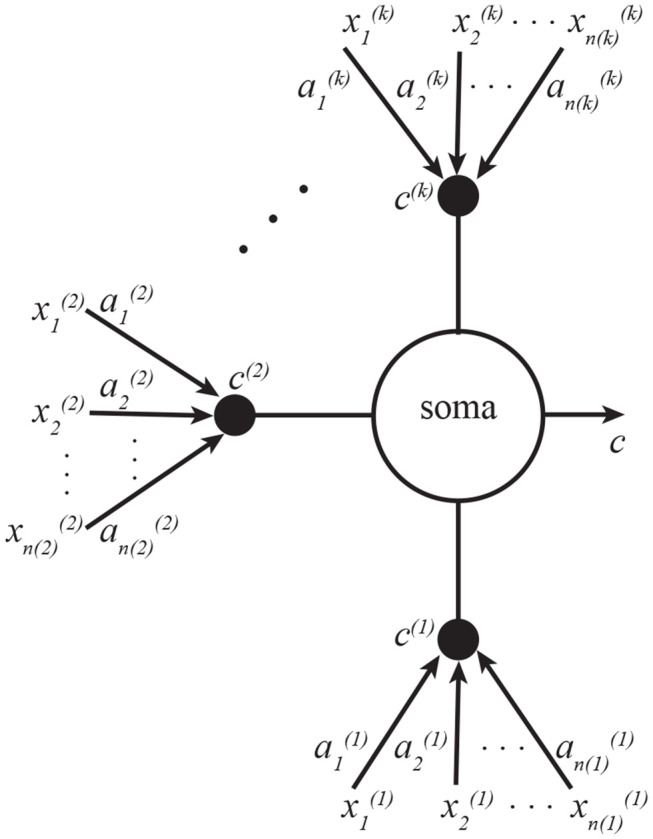
A neuron with *k* dendritic compartments can implement *k*-view CCA. Here the soma sums the currents from all *k* compartments, which are indexed by superscripts. Subscripts in this figure denote indices of vector elements.

This algorithm can be implemented by a neuron with *k* dendritic compartments. The neural structure is depicted in Figure 3, and the pseudocode—in Algorithm 2. As a generalization of the neuron shown in Figure 1, the soma is summing all *k* inputs instead of just two, and each branch of dendrites is carrying out exactly the same learning operations as Figure 1.

**Algorithm 2:** Multiview CCA neuron

**Input:** Parameters $\eta_{a}^{\left(i\right)}$ and $\eta_{\alpha }^{\left(i\right)}$ for *i* = 1, 2, …, *k*. Initial dendritic variables α^(*i*)^ and initial synaptic weights **a**^(*i*)^ ∈ ℝ^*n*^^(*i*)^ *i* = 1, 2, …, *k*. **for** *t* = 1, 2, 3, …, *T* **do** // Neural activity Receive inputs ${\text{x}}_{t}^{\left(i\right)}$ for *i* = 1, 2, …, *k* Calculate dendritic currents for all *k* compartments: $c_{t}^{\left(i\right)}:={{\text{a}}_{t}^{\left(i\right)}}^{⊤}{\text{x}}_{t}^{\left(i\right)}$, *i* = 1, 2, …, *k* Calculate neuronal output: $c_{t}:=\overset{k}{\underset{i=1}{\sum }}c_{t}^{\left(i\right)}$ // Synaptic and homeostatic plasticity Update synaptic weights: ${\text{a}}_{t+1}^{\left(i\right)}={\text{a}}_{t}^{\left(i\right)}+\eta_{a}^{\left(i\right)}\left(c_{t}-\alpha_{t}^{\left(i\right)}c_{t}^{\left(i\right)}\right){\text{x}}_{t}^{\left(i\right)}$, *i* = 1, 2, …, *k* Update dendritic variables: $\alpha_{t+1}^{\left(i\right)}=\alpha_{t}^{\left(i\right)}+\frac{\eta_{\alpha }^{\left(i\right)}}{2}\left({\left(c_{t}^{\left(i\right)}\right)}^{2}-1\right)$, *i* = 1, 2, …, *k* **end for**

## 6. Numerical Simulations

In this section, we provide numerical simulations of our algorithms.

### 6.1. Multichannel CCA Algorithm

We simulated our CCA network (Algorithm 1) and several other algorithms on various datasets.

#### 6.1.1. Datasets

**Figure 4:**
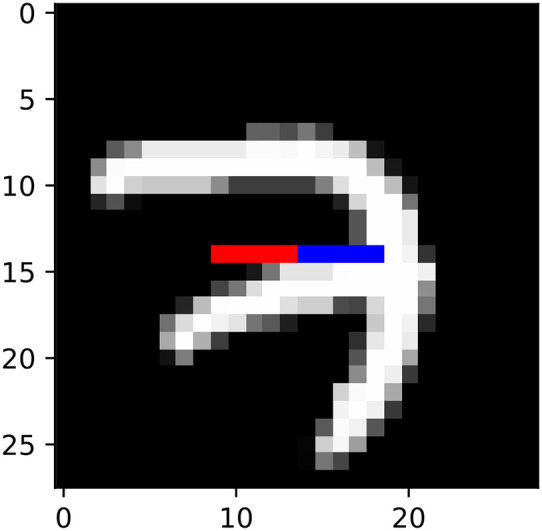
Illustration of mnist datasets. **x**_*t*_ and **y**_*t*_ corresponds to red and blue pixels, respectively.

We use two 5-dimensional inputs, i.e., ${\text{x}}_{t},{\text{y}}_{t}\in {\mathbb{R}}^{5}$, and three datasets, whose samples are generated according to the following procedures:

1. gaussian: We draw **x**_*t*_ and **y**_*t*_ jointly from a 10-dimensional Gaussian distribution with random covariance matrices **A**^⊤^**A**/100, where elements of the square matrix **A** are independently drawn from a standard Gaussian distribution.
2. mnist: We generated a synthetic dataset from the training portion of the MNIST dataset (LeCun, 1998). We took the 15th row of 28-by-28 MNIST images, and set **x**_*t*_ to be the 10th to 14th pixels of this row, and **y**_*t*_ to be the 15th to 19th pixels of this row (Figure 4).
3. mediamill: We used the Mediamill dataset (Snoek et al., 2006) ^1^, which contains 101 textual features and 120 visual features of multiple TV frames. We let **x**_*t*_ be the 5 textual features with highest frequency of occurrence, while **y**_*t*_ is set to be the first 5 visual features in the dataset.

All of the above datasets are centered such that the mean of ${\text{x}}_{t}\in {\mathbb{R}}^{5}$ and ${\text{y}}_{t}\in {\mathbb{R}}^{5}$ are zero. We generate 10,000 samples in advance, and take **x**_*t*_ and **y**_*t*_ from these samples randomly during each training step. We extract the top *d* = 3 canonical components for all datasets.

#### 6.1.2. Metrics of Performance

Next, we define the performance metrics for comparisons between different algorithms.

- **Normalized objective:** The first metric we used is the value of objective function during training divided by the optimal objective *f*(**A**, **B**)/*f*(**A**_*cca*_, **B**_*cca*_) where *f* is defined as:
  (17) $$\begin{array}{l} f(\text{A},\text{B})=\frac{\frac{1}{T}\sum_{i=1}^{d}\sum_{t=1}^{T}\left({\text{a}}_{i}^{⊤}{\text{x}}_{t})({\text{b}}_{i}^{⊤}{\text{y}}_{t}\right)}{{\left[\frac{1}{T}\sum_{i=1}^{d}\sum_{t=1}^{T}{\left({\text{a}}_{i}^{⊤}{\text{x}}_{t}\right)}^{2}\right]}^{1/2}{\left[\frac{1}{T}\sum_{i=1}^{d}\sum_{t=1}^{T}{\left({\text{b}}_{i}^{⊤}{\text{y}}_{t}\right)}^{2}\right]}^{1/2}} \end{array}$$
  Here **A** and **B** are synaptic weights, as in Algorithm 1, and **a**_*i*_ (or **b**_*i*_) is the *i*th column of **A** (or **B**). **A**_*cca*_ and **B**_*cca*_ are the correct solution of CCA; therefore, normalized objective should converge to one if the algorithm is successful. The numerator is the objective of (6), while the denominator corresponds to the constraints. We include the denominator because during the optimization process the constraints are not always fulfilled, potentially causing a misleading normalized objective. Note that this measure can take values above 1 again due to constraints being not satisfied.
- **Angular error:** We used a metric from Ge et al. (2016) defined as:
  (18) $$\begin{array}{l} \text{arccos}\left(\frac{\frac{1}{T}\sum_{i=1}^{d}\sum_{t=1}^{T}\left[\left({\text{a}}_{i}^{⊤}{\text{x}}_{t}\right)\left({\text{a}}_{cca,i}^{⊤}{\text{x}}_{t}\right)+\left({\text{b}}_{i}^{⊤}{\text{y}}_{t}\right)\left({\text{b}}_{cca,i}^{⊤}{\text{y}}_{t}\right)\right]}{{\left\{\frac{1}{T}\sum_{i=1}^{d}\sum_{t=1}^{T}\left[{\left({\text{a}}_{i}^{⊤}{\text{x}}_{t}\right)}^{2}+{\left({\text{b}}_{i}^{⊤}{\text{y}}_{t}\right)}^{2}\right]\right\}}^{1/2}{\left\{\frac{1}{T}\sum_{i=1}^{d}\sum_{t=1}^{T}\left[{\left({\text{a}}_{cca,i}^{⊤}{\text{x}}_{t}\right)}^{2}+{\left({\text{b}}_{cca,i}^{⊤}{\text{y}}_{t}\right)}^{2}\right]\right\}}^{1/2}}\right) \end{array}$$
  where subscript, _*cca*_, denotes correct solution of CCA. Angular error is zero when the algorithm finds the correct solution of CCA. This metric measures the cosine of the angles between (**A**, **B**) and (**A**_*cca*_, **B**_*cca*_) based on the following inner product:
  (19) $$\begin{array}{l} \langle \left(A,B\right),\left(A_{cca},B_{cca}\right)\rangle =\sum_{i=1}^{d}[a_{i}^{⊤}\left(\frac{1}{T}\sum_{t=1}^{T}x_{t}x_{t}^{⊤}\right)a_{cca,i}\\ \;+\;b_{i}^{⊤}\left(\frac{1}{T}\sum_{t=1}^{T}y_{t}y_{t}^{⊤}\right)b_{cca,i}], \end{array}$$
  which gives higher weight to synapses whose input has higher variance.

#### 6.1.3. Simulated Algorithms

We simulated four algorithms:

1. proposed: CCA network (Algorithm 1) with linearly decaying learning rates. We set η = η_*a*_ = η_*b*_ = η_α_ = η_β_ = η_*m*_, and η(*t*) = 0.02 × max(1 − α*t*, 0.1), where α = 5 × 10^−6^. We also tried other learning rate decay schemes such as η(*t*)∝1/*t* or $1/\sqrt{t}$, but found them to be performing worse. We initialized all weights randomly by drawing each of their elements independently from a standard gaussian distribution.
2. nondecay: CCA network(Algorithm 1) with constant learning rate. We used the same parametrization as above but with α = 0.
3. nonlocal: The nonlocal (biologically implausible) algorithm (Algorithm 3). The initialization of weights and initial learning rates are exactly the same as Algorithm 1, with constant learning rate during training.
4. MSG-CCA: A non-neural online algorithm “Matrix Stochastic Gradient for CCA” described in Arora et al. (2017). As suggested by Arora et al. (2017), we used learning rate decay scheme $\eta_{t}=\frac{0.1}{\sqrt{t}}$. This algorithm requires an auxiliary training data, i.e., it has to take some samples in advance and learn on them in an offline manner. We chose this sample size to be 100.

#### 6.1.4. Results

**Figure 5:**
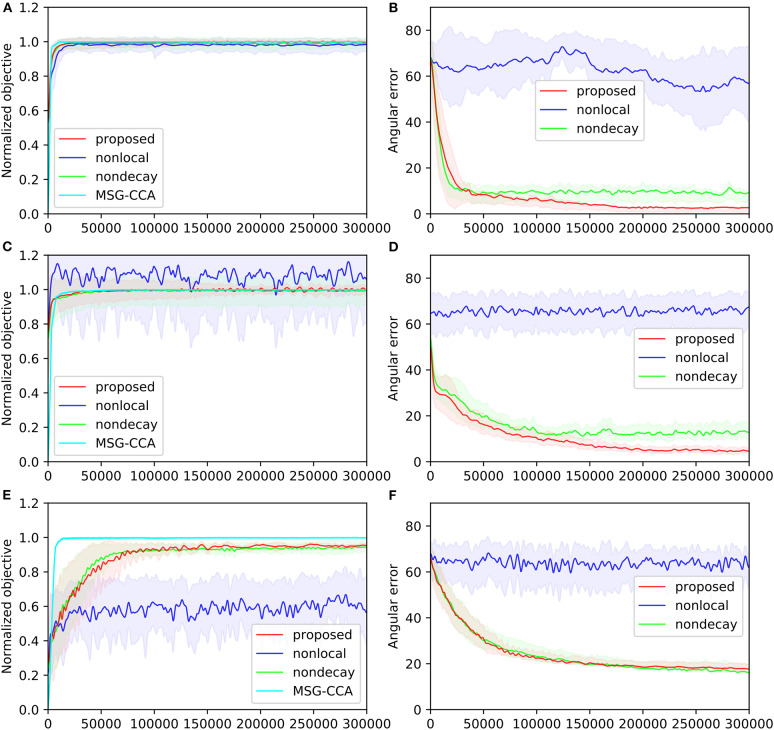
Four algorithms [pyramidal, nonlocal, nondecay, MSG-CCA] run on three datasets [gaussian**(A,B)**, mnist**(C,D)**, mediamill**(E,F)**]. Performance is measured by normalized objective **(A,C,E)** and angular error **(B,D,F)** All algorithms are run 10 times except MSG-CCA because of computational constraints. Solid lines show mean value over 10 simulations. Shaded regions of the same color show standard deviation over 10 simulations. Both mean and standard deviation are smoothed along the time course of training.

The performances of all four algorithms [pyramidal, nonlocal, nondecay, MSG-CCA] on the three datasets are shown in Figure 5. The angular error of MSG-CCA algorithm is not calculated because the algorithm does not produce a deterministic and explicit estimate of the canonical variables (Arora et al., 2012), however calculating normalized objective is possible. All algorithms have similar performance except the nonlocal algorithm which does not converge at all. Note that the normalized objective can go over 1 because the constraint of CCA may not be satisfied. The proposed and nondecay algorithms only differ in their learning rate scheme, and we can see that the proposed algorithm with learning rate decay has a better performance.

### 6.2. Multiview CCA Algorithm

Next, we simulated our Algorithm 2 for multiview CCA. We set constant learning rates $\eta_{a}^{\left(i\right)}=\eta_{\alpha }^{\left(i\right)}=0.005$ for all *i* = 1, 2, ⋯ , *k*, and initialize all network weights according to standard gaussian distribution.

#### 6.2.1. Datasets

**Figure 6:**
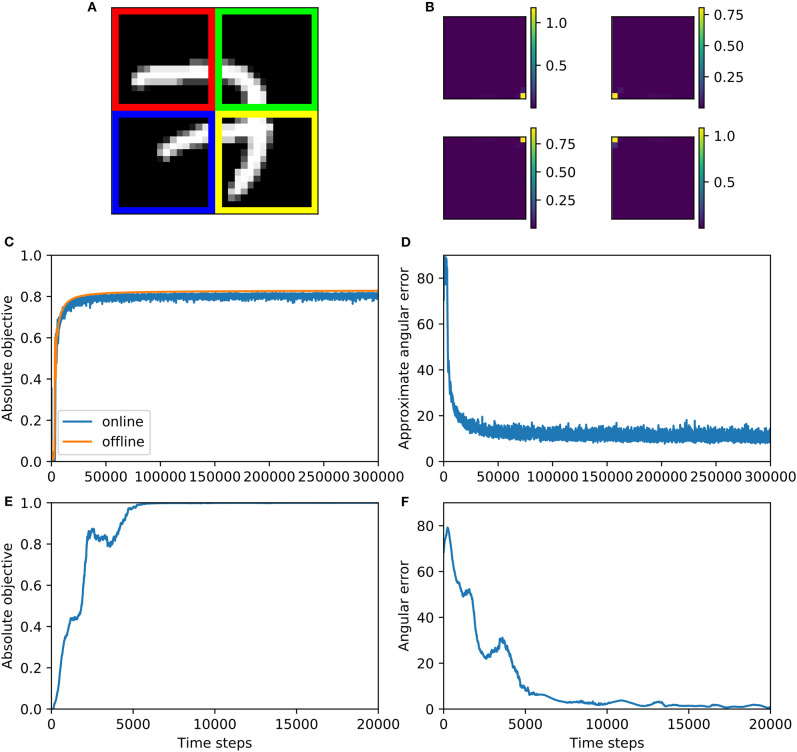
Multiview CCA algorithm 2 is trained on mnist4X dataset **(A–D)** and svd-max dataset **(E,F)**. **(A)** This figure shows how MNIST dataset is split, where red, green, blue, and yellow parts of the image correspond to ${\text{x}}_{t}^{\left(1\right)}$, ${\text{x}}_{t}^{\left(2\right)}$, ${\text{x}}_{t}^{\left(3\right)}$, and ${\text{x}}_{t}^{\left(4\right)}$, respectively. **(B)** The synaptic weights after training, which are measured by variance contribution, match our expectation for groundtruth. The variance contribution is calculated by $u_{i}=\frac{1}{T}\overset{T}{\underset{t=1}{\sum }}{\left(a_{i}x_{i,t}\right)}^{2}$ where *i* is the index of image pixel. In this visualization, these *u*_*i*_ are positioned according to the positions of their corresponding pixel and their values are indicated by the colorbars. **(C)** Absolute objective for both online and offline version of Algorithm 2 increases during training and converge to near 0.8. **(D)** Approximate angular error decreases and then fluctuate above zero during online training. **(E)** Objective function increases during training and converge to one—the largest possible correlation value. **(F)** Angular error decreases during training and eventually fluctuate around zero.

Since multiview CCA does not have an analytical solution (Kettenring, 1971), and is computationally intractable (Rupnik and Shawe-Taylor, 2010), we choose datasets to which we know or approximately know the solution. The following two datasets are used:

1. mnist4X: Here we set ${\text{x}}_{t}^{\left(1\right)},{\text{x}}_{t}^{\left(2\right)},{\text{x}}_{t}^{\left(3\right)},{\text{x}}_{t}^{\left(4\right)}$ to be four quarters of the *t*'th MNIST image (Figure 6A). The closer two pixels are, the higher the correlation between them is, so our generalized CCA would extract pixels closest to each other. In this case, it would approximately be the four pixels at the center of the whole image. Like before, instead of drawing a sample at every training step, we take 10000 MNIST images and generate from them 10,000 samples of ${\text{x}}_{t}^{\left(1\right)},{\text{x}}_{t}^{\left(2\right)},{\text{x}}_{t}^{\left(3\right)},{\text{x}}_{t}^{\left(4\right)}$ in advance, and randomly draw a sample of these at each training step.
2. svd-max: We used singular value decomposition (SVD) to construct a dataset such that all pairs of dendritic currents (that is *c*^(*i*)^, *i* = 1, 2, ⋯ , *k*) have a correlation coefficient of one, i.e. maximally correlated. Specifically, we let $\left({\text{x}}_{1}^{\left(i\right)},{\text{x}}_{2}^{\left(i\right)},\cdots \;,{\text{x}}_{T}^{\left(i\right)}\right)={\text{X}}^{\left(i\right)}={\text{U}}^{\left(i\right)}{\text{S}}^{\left(i\right)}{{\text{V}}^{\left(i\right)}}^{⊤}$, where columns of **U**^(*i*)^ ∈ ℝ^*n*^^(*i*)^ × *n*^(*i*)^ (and also **V**^(*i*)^ ∈ ℝ*T* × ^*n*^^(*i*)^) are orthonormal to each other, and **S**^(*i*)^ ∈ ℝ^*n*^^(*i*)^ × *n*^(*i*)^ is diagonal. For all *i* = 1, 2, ⋯ , *k*, diagonal elements of **S**^(*i*)^ are independently drawn from *Uniform*([0.1, 1]), and then sorted in descending order. For all *i* = 1, 2, ⋯ , *k*, **U**^(*i*)^ is constructed by perfroming SVD on a standard gaussian distributed matrix of the same size. The first column of **V**^(1)^, **V**^(2)^, ⋯ , **V**^(*k*)^ are set to be the same, and all the other columns of all these *k* matrices are set to be orthonormal to each other: this is done by first constructing a $T\times \left(\overset{k}{\underset{i=1}{\sum }}n^{\left(i\right)}-k+1\right)$ matrix whose columns are orthonormal to each other, and then slicing this matrix to yield **V**^(1)^, **V**^(2)^, ⋯ , **V**^(*k*)^. The weights that optimize objective function (13) is ${\text{a}}^{\left(i\right)}=\frac{1}{s_{1}^{\left(i\right)}}{\text{u}}_{1}^{\left(i\right)}$, where $s_{1}^{\left(i\right)}$ is the first element of **S**^(*i*)^, and ${\text{u}}_{1}^{\left(i\right)}$ is the first column of **U**^(*i*)^. For the simulation below, we set *k* = 3, *n*^(1)^ = 4, *n*^(2)^ = 5, *n*^(3)^ = 6, *T* = 10, 000.

#### 6.2.2. Metrics of Performance

As before, we have to define the metrics for performance of multiview CCA algorithm.

- **Absolute objective:**
  (20) $$\begin{array}{l} \frac{2}{k\left(k-1\right)}\overset{k}{\underset{i=1}{\sum }}\overset{k}{\underset{j=i+1}{\sum }}\frac{\frac{1}{T}\sum_{t=1}^{T}\left({{\text{a}}^{\left(i\right)}}^{⊤}{\text{x}}_{t}\right)\left({{\text{a}}^{\left(j\right)}}^{⊤}{\text{x}}_{t}\right)}{{\left[\frac{1}{T}\sum_{t=1}^{T}{\left({{\text{a}}^{\left(i\right)}}^{⊤}{\text{x}}_{t}\right)}^{2}\right]}^{1/2}{\left[\frac{1}{T}\sum_{t=1}^{T}{\left({{\text{a}}^{\left(j\right)}}^{⊤}{\text{x}}_{t}\right)}^{2}\right]}^{1/2}}, \end{array}$$
  which is strictly between −1 and 1.
- **Angular error:**
  (21) $$\begin{array}{l} \arccos\left(\frac{\frac{1}{T}\sum_{t=1}^{T}\sum_{i=1}^{k}\left({{\text{a}}^{\left(i\right)}}^{⊤}{\text{x}}_{t}\right)\left({{\text{a}}_{cca}^{\left(i\right)}}^{⊤}{\text{x}}_{t}\right)}{{\left[\frac{1}{T}\sum_{t=1}^{T}\sum_{i=1}^{k}{\left({{\text{a}}^{\left(i\right)}}^{⊤}{\text{x}}_{t}\right)}^{2}\right]}^{1/2}{\left[\frac{1}{T}\sum_{t=1}^{T}\sum_{i=1}^{d}{\left({{\text{a}}_{cca}^{\left(i\right)}}^{⊤}{\text{x}}_{t}\right)}^{2}\right]}^{1/2}}\right), \end{array}$$
  where _*cca*_ denotes correct solution to CCA.

#### 6.2.3. Results

The performance of the algorithm on the mnist4X dataset is shown in Figures 6A–D. We visualize the learned weights on the mnist4X dataset (Figure 6B) and observe that all the weights cluster in the center of the MNIST image. Absolute objective and angular error are shown in Figures 6C,D. To calculate a groundtruth value of optimal weights, we ran the offline version of algorithm 2 on mnist4X dataset. We used the offline weights to approximate the groundtruth weights, and calculated an approximated angular error (Figure 6D). Training results on svd-max dataset is shown in Figures 6E,F. Note that for this dataset the analytical solution is available, so we could calculate angular error precisely.

## 7. Conclusion

In this paper, we propose mathematically tractable multicompartment neuron models that capture more biological features than previous such models. A multichannel CCA algorithm is implemented by an asymmetric network of two-compartment pyramidal neurons. A neuron with more than two dendritic compartments may implement an online multiview CCA algorithm.

Naturally, our model is a drastic simplification of a real biological pyramidal neuron. We assumed that the activities of neurons are linear, continuous and deterministic, instead of nonlinear, spiking and stochastic. The two-compartment structure is the most prominent feature of pyramidal neuron's dendritic structure, which is well-captured by our model, but this is also a simplification of actual dendritic integration, which depends on more complex dendritic morphology (Spruston, 2008). In our model, distal and proximal compartments have symmetric status in driving neuronal activity and learning rules, since the two input vectors have symmetric status in CCA. However, in a biological pyramidal neuron, these two compartments are asymmetric: their sizes are different, and, for example, their excitability might also be different (Spruston, 2008). Dendritic and synaptic nonlinearities which we ignored can endow a neuron with a rich computational repertoire (Poirazi and Mel, 2001; Poirazi et al., 2003; Polsky et al., 2004; Larkum, 2013; Jadi et al., 2014; Haga and Fukai, 2017). Temporal dynamics and synaptic activity delays are other aspects not captured by our model.

Pyramidal neurons have been proposed to integrate feedback stimulation in the distal dendrite and feedforward information in the proximal dendrite (Spratling and Johnson, 2004). Since our model of pyramidal neurons performs CCA on distal and proximal inputs, it provides a new interpretation of the computation performed on feedforward and feedback inputs. Some recent work (Guerguiev et al., 2017; Sacramento et al., 2018) proposes that top-down dendritic input may be instructively gating plasticity in feedforward synapses, implementing approximately the backpropagation algorithm. Our model may provide an alternative mechanism for credit assignment, where a correlation between error related feedback signals and feedforward signals are learned.

It has been proposed that multisensory integration requires correlation detection among different sensory modalities (Parise and Ernst, 2016), and that such integration could happen on a single neuron (Stein and Stanford, 2008). The multi-compartmental version of our algorithm that performs multiview CCA can be useful for modeling such a neuron, which takes signals from different modalities as inputs and extracts a component from each input that are maximally correlated with each other. One experimental observation that may fit such interpretation is that correlated sensory and motor information has been shown to be detected by pyramidal neurons through dendritic integration (Xu et al., 2012).

## Data Availability Statement

The datasets generated for this study are available on request to the corresponding author.

## Author Contributions

All authors designed the study. CP, XZ, and DC contributed to the analytical results and wrote the manuscript. XZ performed the numerical simulations.

## Conflict of Interest

The authors declare that the research was conducted in the absence of any commercial or financial relationships that could be construed as a potential conflict of interest.

## Footnotes

^1^downloaded from http://isis-data.science.uva.nl/cgmsnoek/mediamill/mediamill-challenge.tar.gz/
