# Supplementary material for: Neurons as Canonical Correlation Analyzers
Source: Front Comput Neurosci. 2020 Jun 30;14:55. doi: 10.3389/fncom.2020.00055 (PMC7338892; doi:10.3389/fncom.2020.00055)
Supplement: Supplementary file 1 [file Data_Sheet_1.pdf]

Supplementary Information

A Solution to CCA and its properties

Define covariance matrices:

$$\mathbf{\Sigma}_{xx} = \frac{1}{T}\sum_{t=1}^{T}\mathbf{x}_t\mathbf{x}_t^\top, \qquad \mathbf{\Sigma}_{xy} = \frac{1}{T}\sum_{t=1}^{T}\mathbf{x}_t\mathbf{y}_t^\top, \qquad \mathbf{\Sigma}_{yy} = \frac{1}{T}\sum_{t=1}^{T}\mathbf{y}_t\mathbf{y}_t^\top \tag{22}$$

The solution of CCA is obtained through singular value decomposition [15]:

$$\begin{aligned}
&\mathbf{\Sigma}_{xx}^{-1/2}\mathbf{\Sigma}_{xy}\mathbf{\Sigma}_{yy}^{-1/2} = \tilde{\mathbf{a}}\mathbf{S}\tilde{\mathbf{b}}^\top \\
&(\mathbf{a}_1, \mathbf{a}_2, \cdots, \mathbf{a}_m) = \mathbf{a} = \mathbf{\Sigma}_{xx}^{-1/2}\tilde{\mathbf{a}} \\
&(\mathbf{b}_1, \mathbf{b}_2, \cdots, \mathbf{b}_n) = \mathbf{b} = \mathbf{\Sigma}_{yy}^{-1/2}\tilde{\mathbf{b}} \\
&\mathbf{S} = \mathrm{diag}(\sigma_1, \sigma_2, \cdots, \sigma_{\min(m,n)}) \in \mathbb{R}^{m\times n}
\end{aligned} \tag{23}$$

where the solution is $(\mathbf{a}_1, \mathbf{a}_2, \cdots, \mathbf{a}_d)$ and $(\mathbf{b}_1, \mathbf{b}_2, \cdots, \mathbf{b}_d)$ with d neurons. Note that $\{\mathbf{a}_1, \mathbf{a}_2, \cdots, \mathbf{a}_m\}$ and $\{\mathbf{b}_1, \mathbf{b}_2, \cdots, \mathbf{b}_n\}$ are complete bases in $\mathbb{R}^m$ and $\mathbb{R}^n$, respectively. Therefore, we can express arbitrary vectors as the linear combination of them, a property that we will often use in proofs.

The following properties hold for the solution to CCA [15]:

$$\begin{aligned}
&\mathbf{a}_i^T\mathbf{\Sigma}_{xx}\mathbf{a}_j = \delta_{ij} \\
&\mathbf{b}_i^T\mathbf{\Sigma}_{yy}\mathbf{b}_j = \delta_{ij} \\
&\mathbf{a}_i^T\mathbf{\Sigma}_{xy}\mathbf{b}_j = \delta_{ij}\sigma_i \\
&1 \geq \sigma_1 \geq \sigma_2 \geq \cdots \geq \sigma_{\min(m,n)} \geq 0 \\
&\mathbf{\Sigma}_{xy}\mathbf{b}_i = \sigma_i\mathbf{\Sigma}_{xx}\mathbf{a}_i, \quad \mathbf{\Sigma}_{yx}\mathbf{a}_i = \sigma_i\mathbf{\Sigma}_{yy}\mathbf{b}_i
\end{aligned} \tag{24}$$

B Linear stability analysis for single-channel CCA

Here, we show that the fixed point of the single-channel CCA algorithm corresponds to the solution of Eq. (1). Using a stochastic approximation, we analyze the linear stability of our algorithm and show that the CCA solution is linearly stable. In the limit of small learning rates, the evolution of the single-channel CCA

algorithm (Eq. (5)) can be approximated by a dynamical system [44] (we use symbols defined in SI A):

$$\begin{aligned}
\frac{d\mathbf{a}}{dt} &= \eta_a \left[(1-\alpha)\mathbf{\Sigma}_{xx}\mathbf{a} + \mathbf{\Sigma}_{xy}\mathbf{b}\right], \\
\frac{d\mathbf{b}}{dt} &= \eta_b \left[\mathbf{\Sigma}_{yx}\mathbf{a} + (1-\beta)\mathbf{\Sigma}_{yy}\mathbf{b}\right], \\
\frac{d\alpha}{dt} &= \frac{\eta_\alpha}{2}\left(\mathbf{a}^\top \mathbf{\Sigma}_{xx}\mathbf{a} - 1\right), \\
\frac{d\beta}{dt} &= \frac{\eta_\beta}{2}\left(\mathbf{b}^\top \mathbf{\Sigma}_{yy}\mathbf{b} - 1\right),
\end{aligned} \tag{25}$$

where $\mathbf{a}, \mathbf{x} \in \mathbb{R}^{m\times 1}$, $\mathbf{b}, \mathbf{y} \in \mathbb{R}^{n\times 1}$, and $\mathbf{\Sigma}_{xy}$ denotes the covariance matrix between random vectors $\mathbf{x}$ and $\mathbf{y}$.

Proposition 2. *Principal canonical variables are stable fixed points of the dynamical system (25), if the following two conditions hold:*

1. $\eta_a = \eta_b$,
2. $\nexists l_a = \frac{\eta_\alpha}{\eta_\beta} l_b > 0,\ s.t.\ \mathbf{\Sigma}_{xx}\mathbf{a}_1 = l_a\mathbf{a}_1, \mathbf{\Sigma}_{yy}\mathbf{b}_1 = l_b\mathbf{b}_1$.

Before proving the proposition, we comment on the second condition above which rules out an exceptional case. Since $\mathbf{a}_1$ is an eigenvector of $\mathbf{\Sigma}_{xx}^{-1}\mathbf{\Sigma}_{xy}\mathbf{\Sigma}_{yy}^{-1}\mathbf{\Sigma}_{yx}$, it is generally not an eigenvector of $\mathbf{\Sigma}_{xx}$. For the same reason, $\mathbf{b}_1$ is generally not an eigenvector of $\mathbf{\Sigma}_{yy}$. Even if they both are, there only exist one ratio of learning rates $\frac{\eta_\alpha}{\eta_\beta}$ such that second condition is true. Therefore, the condition of Proposition 2 is almost surely satisfied.

Proof. We will prove the proposition by explicitly calculating the Jacobian of the dynamical system at the fixed point and showing that it is negative definite.

We first calculate all the fixed points of Eq. (25), and show that they are given by the canonical variables. Fixed points satisfy:

$$\begin{cases}
(1-\alpha)\mathbf{\Sigma}_{xx}\mathbf{a} + \mathbf{\Sigma}_{xy}\mathbf{b} = 0 \\
\mathbf{\Sigma}_{yx}\mathbf{a} + (1-\beta)\mathbf{\Sigma}_{yy}\mathbf{b} = 0 \\
\frac{1}{2}(\mathbf{a}^\top \mathbf{\Sigma}_{xx}\mathbf{a} - 1) = 0 \\
\frac{1}{2}(\mathbf{b}^\top \mathbf{\Sigma}_{yy}\mathbf{b} - 1) = 0
\end{cases} \tag{26}$$

We expand $\mathbf{a}$ and $\mathbf{b}$ in the CCA bases:

$$\mathbf{a} = \sum_{i=1}^{n} \alpha_i \mathbf{a}_i, \qquad \mathbf{b} = \sum_{i=1}^{m} \beta_i \mathbf{b}_i \tag{27}$$

Using properties shown in SI A we have:

$$\begin{cases} \alpha = \beta = \mathbf{a}^\top \mathbf{\Sigma}_{xy} \mathbf{b} + 1 \\ \sum_{i=1}^{n} (1-\alpha)\alpha_i \mathbf{\Sigma}_{xx} \mathbf{a}_i + \sum_{i=1}^{m} \beta_i \sigma_i \mathbf{\Sigma}_{xx} \mathbf{a}_i = 0 \\ \sum_{i=1}^{n} \alpha_i \sigma_i \mathbf{\Sigma}_{yy} \mathbf{b}_i + \sum_{i=1}^{m} (1-\beta)\beta_i \mathbf{\Sigma}_{yy} \mathbf{b}_i = 0 \\ \sum_{i=1}^{n} \alpha_i^2 = 1 \\ \sum_{i=1}^{m} \beta_i^2 = 1 \end{cases} \tag{28}$$

As all $\mathbf{\Sigma}_{xx}\mathbf{a}_i$ are linearly independent, the following holds true for $i = 1, 2, \cdots, \min(m, n)$:

$$\begin{cases} (1-\alpha)\alpha_i + \sigma_i \beta_i = 0 \\ \sigma_i \alpha_i + (1-\beta)\beta_i = 0 \end{cases} \tag{29}$$

And when $i > \min(m, n)$ every α_i or β_i is zero. From this, we obtain the following condition for $i = 1, 2, \cdots, \min(m, n)$:

$$\begin{vmatrix} 1-\alpha & \sigma_i \\ \sigma_i & 1-\beta \end{vmatrix} = 0 \text{ is a necessary condition of } \alpha_i^2 + \beta_i^2 > 0 \tag{30}$$

Assuming all σ_i's are not equal, i.e. $\sigma_1 > \sigma_2 > \cdots > \sigma_{\min(m,n)}$, at most one i let condition (30) be satisfied. When i does satisfy (30), we must have $\mathbf{a} = \pm\mathbf{a}_i$ and $\mathbf{b} = \pm\mathbf{b}_i$. Thus, system (25) have $4\min(n, m)$ fixed points, corresponding to the canonical variable pairs up to a sign. For each fixed point, we calculate α and β:

$$\alpha = \beta = \mathbf{a}^\top \mathbf{\Sigma}_{xy} \mathbf{b} + 1 = 1 + \mathrm{sgn}(\mathbf{a}^\top \mathbf{a}_i \mathbf{b}^\top \mathbf{b}_i)\sigma_i, \tag{31}$$

where sgn is the sign function.

Given the fixed points of system (25), we can now perform a linear stability analysis. By rescaling

$\hat{\alpha} = \sqrt{\frac{\eta_a}{\eta_\alpha}}\alpha$ and $\hat{\beta} = \sqrt{\frac{\eta_b}{\eta_\beta}}\beta$ we get:

$$\begin{aligned} \frac{d\mathbf{a}}{dt} &= \eta_a \left[\left(1 - \sqrt{\frac{\eta_\alpha}{\eta_a}}\hat{\alpha}\right) \mathbf{\Sigma}_{xx}\mathbf{a} + \mathbf{\Sigma}_{xy}\mathbf{b} \right], \\ \frac{d\mathbf{b}}{dt} &= \eta_b \left[\mathbf{\Sigma}_{yx}\mathbf{a} + \left(1 - \sqrt{\frac{\eta_\beta}{\eta_b}}\hat{\beta}\right) \mathbf{\Sigma}_{yy}\mathbf{b} \right], \\ \frac{d\hat{\alpha}}{dt} &= \frac{\eta_\alpha}{2}\sqrt{\frac{\eta_a}{\eta_\alpha}} \left(\mathbf{a}^\top \mathbf{\Sigma}_{xx}\mathbf{a} - 1\right), \\ \frac{d\hat{\beta}}{dt} &= \frac{\eta_\beta}{2}\sqrt{\frac{\eta_b}{\eta_\beta}} \left(\mathbf{b}^\top \mathbf{\Sigma}_{yy}\mathbf{b} - 1\right), \end{aligned} \tag{32}$$

Focusing on the top canonical variables (i.e. solution to (1)),

$$\mathbf{a} = \pm\mathbf{a}_1, \qquad \mathbf{b} = \mathrm{sgn}(\mathbf{a}^\top\mathbf{a}_1)\mathbf{b}_1, \qquad \hat{\alpha} = \sqrt{\frac{\eta_a}{\eta_\alpha}}(\sigma_1 + 1), \qquad \hat{\beta} = \sqrt{\frac{\eta_b}{\eta_\beta}}(\sigma_1 + 1), \tag{33}$$

we get the Jacobian matrix at the fixed points:

$$\mathbf{J} = \begin{bmatrix} -\eta_a\sigma_1\mathbf{\Sigma}_{xx} & \eta_a\mathbf{\Sigma}_{xy} & \mp\sqrt{\eta_a\eta_\alpha}\mathbf{\Sigma}_{xx}\mathbf{a}_1 & 0 \\ \eta_b\mathbf{\Sigma}_{yx} & -\eta_b\sigma_1\mathbf{\Sigma}_{yy} & 0 & \mp\sqrt{\eta_b\eta_\beta}\mathbf{\Sigma}_{yy}\mathbf{b}_1 \\ \pm\sqrt{\eta_a\eta_\alpha}\mathbf{a}_1^\top\mathbf{\Sigma}_{xx} & 0 & 0 & 0 \\ 0 & \pm\sqrt{\eta_b\eta_\beta}\mathbf{b}_1^\top\mathbf{\Sigma}_{yy} & 0 & 0 \end{bmatrix}. \tag{34}$$

Next, we show that the Jacobian matrix is negative definite, which means $\mathbf{u}^\top\mathbf{J}\mathbf{u} < 0$ for any nonnegative $m + n + 2$ dimensional vector $\mathbf{u}$:

$$\mathbf{u} = \begin{bmatrix} \mathbf{w} \\ \mathbf{v} \\ \lambda \\ \gamma \end{bmatrix}, \quad \text{where } \mathbf{w} \in \mathbb{R}^{m\times 1}, \mathbf{v} \in \mathbb{R}^{n\times 1}, \tag{35}$$

We can expand $\mathbf{w}$ and $\mathbf{v}$ in CCA bases:

$$\mathbf{w} = \sum_{i=1}^{m} \alpha_i\mathbf{a}_i, \qquad \mathbf{v} = \sum_{i=1}^{n} \beta_i\mathbf{b}_i \tag{36}$$

If $\eta_a = \eta_b$, $\mathbf{J}$ is negative definite:

$$\begin{aligned}\mathbf{u}^\top \mathbf{J}\mathbf{u} &= \eta_a \left[-\sigma_1(\mathbf{w}^\top \mathbf{\Sigma}_{xx}\mathbf{w} + \mathbf{v}^\top \mathbf{\Sigma}_{yy}\mathbf{v}) + 2\mathbf{w}^\top \mathbf{\Sigma}_{xy}\mathbf{v}\right] \\ &= \eta_a \left[-\sigma_1 \sum_{i=1}^{m} \alpha_i^2 - \sigma_1 \sum_{i=1}^{n} \beta_i^2 + 2 \sum_{i=1}^{\min(m,n)} \sigma_i \alpha_i \beta_i\right] \\ &\leq \eta_a \left[-\sigma_1 \sum_{i=1}^{\min(m,n)} \left(\alpha_i^2 + \beta_i^2 - 2\frac{\sigma_i}{\sigma_1}\alpha_i\beta_i\right)\right]. \end{aligned} \tag{37}$$

The condition for $\alpha_i^2 + \beta_i^2 - 2\frac{\sigma_i}{\sigma_1}\alpha_i\beta_i$ to always be positive (except when $\alpha_i = \beta_i = 0$) is $\Delta = 4(\frac{\sigma_i}{\sigma_1})^2 - 4 < 0$. Since all $\sigma_i \leq \sigma_1$, this condition holds true for all i. Therefore, $\mathbf{u}^\top \mathbf{J}\mathbf{u}$ is always non-positive, which means $\mathbf{J}$ is negative semi-definite. This implies that all eigenvalues of $\mathbf{J}$ has a non-positive real part (statement 1.0.3.1 in [45]). The only possibility for $\mathbf{u}^\top \mathbf{J}\mathbf{u} = 0$ is $\alpha_1 = \beta_1$, and all other $\alpha_i = \beta_i = 0$.

However, for stability, we need a negative definite Jacobian for which we need to rule out a purely imaginary eigenvalue. Suppose we have such an eigenvalue: $\mathbf{J}\mathbf{z} = \zeta\mathbf{z}$. It is not hard to show that both real and imaginary part of $\mathbf{z}$ have to satisfy $(\cdot)^\top \mathbf{J}(\cdot) = 0$, so both of them (and therefore $\mathbf{z}$ itself) can be written as a multiple of the following form:

$$\mathbf{z} = \begin{bmatrix} \mathbf{a}_1 \\ \mathbf{b}_1 \\ \lambda \\ \gamma \end{bmatrix}, \tag{38}$$

where λ and γ can be complex numbers. Without loss of generality, we assume $\mathbf{z}$ takes exactly the above form. Then:

$$\begin{bmatrix} \zeta\mathbf{a}_1 \\ \zeta\mathbf{b}_1 \\ \zeta\lambda \\ \zeta\gamma \end{bmatrix} = \mathbf{J}\mathbf{z} = \pm\sqrt{\eta_a} \begin{bmatrix} -\lambda\sqrt{\eta_\alpha}\mathbf{\Sigma}_{xx}\mathbf{a}_1 \\ -\gamma\sqrt{\eta_\beta}\mathbf{\Sigma}_{yy}\mathbf{b}_1 \\ \sqrt{\eta_\alpha} \\ \sqrt{\eta_\beta} \end{bmatrix} \tag{39}$$

This is possible if and only if $\exists l_a = \frac{\eta_\alpha}{\eta_\beta} l_b > 0,$ s.t. $\mathbf{\Sigma}_{xx}\mathbf{a}_1 = l_a\mathbf{a}_1, \mathbf{\Sigma}_{yy}\mathbf{b}_1 = l_b\mathbf{b}_1$. Since this is ruled out in the statement of this proposition, we conclude that the system of ODEs (25) is stable at the correct solutions to CCA. □

C Online algorithm derived from multichannel CCA

We solve (7) with stochastic gradient steps, which leads to an online algorithm. Defining

$$c^b_{i,t} := \mathbf{a}_{i,t}^\top \mathbf{x}_t, \qquad c^a_{i,t} := \mathbf{b}_{i,t}^\top \mathbf{y}_t, \qquad c_{i,t} := c^b_{i,t} + c^a_{i,t}, \qquad i = 1, \ldots, d \tag{40}$$

we get

$$\begin{aligned}
\mathbf{a}_{i,t+1} &= \mathbf{a}_{i,t} + \eta_a \mathbf{x}_t \left(c_{i,t} - \alpha_{i,t} c^b_{i,t} - \sum_{j=1, j\neq i}^{d} \alpha_{ij,t} c^b_{j,t} \right), \\
\mathbf{b}_{i,t+1} &= \mathbf{b}_{i,t} + \eta_b \mathbf{y}_t \left(c_{i,t} - \beta_{i,t} c^a_{i,t} - \sum_{j=1, j\neq i}^{d} \beta_{ij,t} c^a_{j,t} \right), \\
\alpha_{i,t+1} &= \alpha_{i,t} + \frac{\eta_\alpha}{2} \left({c^b_{i,t}}^2 - 1 \right), \qquad \beta_{i,t+1} = \beta_{i,t} + \frac{\eta_\beta}{2} \left({c^a_{i,t}}^2 - 1 \right), \qquad i = 1, \ldots, d \\
A_{ij,t+1} &= A_{ij,t+1} + \frac{\eta_A}{2} c^b_{i,t} c^b_{j,t}, \qquad B_{ij,t+1} = B_{ij,t+1} + \frac{\eta_B}{2} c^a_{i,t} c^a_{j,t}, \qquad i, j = 1, \ldots, d,\ i \neq j.
\end{aligned} \tag{41}$$

The pseudocode for this nonlocal CCA algorithm is given in algorithm 3.

Algorithm 3 Nonlocal CCA network

Input: Parameters d, η_a, η_b, η_α, η_β, η_A and η_B. Initial $\alpha_1, \ldots, \alpha_d$ and $\beta_1, \ldots, \beta_d$. Initial synaptic weights $\mathbf{a} \in \mathbb{R}^{n\times d}$, $B \in \mathbb{R}^{m\times d}$, and $\boldsymbol{A}, \boldsymbol{B} \in \mathbb{R}^{d\times d}$, where $\boldsymbol{A}, \boldsymbol{B}$ are symmetric and have zero diagonal values.

for $t = 1, 2, 3, \ldots, T$ **do**

`// Neural activity`

Take $\mathbf{x}_t$ and $\mathbf{y}_t$ as input

for $i = 1, \ldots, d$ **do**

Calculate proximal and distal dendritic currents: $c^b_{i,t} = \sum_{j=1}^{n} W_{ji,t} x_{j,t}, \quad c^a_{j,t} = \sum_{j=1}^{m} V_{ji,t} y_{j,t}$,

Calculate pyramidal neuron outputs: $c_{i,t} = c^b_{i,t} + c^a_{i,t}$

end for

`// Synaptic and homeostatic plasticity`

Update synaptic weights:

$\mathbf{a}_{i,t+1} = \mathbf{a}_{i,t} + \eta_a \mathbf{x}_t \left(c_{i,t} - \alpha_{i,t} c^b_{i,t} - \sum_{j=1, j\neq i}^{d} A_{ij,t} c^b_{j,t} \right), \quad i = 1, \ldots, d$

$\mathbf{b}_{i,t+1} = \mathbf{b}_{i,t} + \eta_b \mathbf{y}_t \left(c_{i,t} - \beta_{i,t} c^a_{i,t} - \sum_{j=1, j\neq i}^{d} \beta_{ij,t} c^a_{j,t} \right), \quad i = 1, \ldots, d$

$A_{ij,t+1} = A_{ij,t+1} + \frac{\eta_A}{2} c^b_{i,t} c^b_{j,t}, \qquad B_{ij,t+1} = B_{ij,t+1} + \frac{\eta_B}{2} c^a_{i,t} c^a_{j,t}, \qquad i, j = 1, \ldots, d,\ i \neq j$

Update dendritic variables:

$\alpha_{i,t+1} = \alpha_{i,t} + \frac{\eta_\alpha}{2} \left({c^b_{i,t}}^2 - 1 \right), \quad i = 1, \ldots, d$

$\beta_{i,t+1} = \beta_{i,t} + \frac{\eta_\beta}{2} \left({c^a_{i,t}}^2 - 1 \right), \quad i = 1, \ldots, d$

end for

D Proof of Proposition 1

Proof. For notational ease we define the following correlation matrices:

$$\mathbf{\Sigma}_{xx} = \frac{1}{T}\sum_{t=1}^{T}\mathbf{x}_t\mathbf{x}_t^\top, \qquad \mathbf{\Sigma}_{xy} = \frac{1}{T}\sum_{t=1}^{T}\mathbf{x}_t\mathbf{y}_t^\top, \qquad \mathbf{\Sigma}_{yy} = \frac{1}{T}\sum_{t=1}^{T}\mathbf{y}_t\mathbf{y}_t^\top \tag{42}$$

Note that there are $\min(n, m)$ canonical variables. These satisfy,

$$\mathbf{a}_i^\top\mathbf{\Sigma}_{xx}\mathbf{a}_j = \delta_{ij}, \qquad \mathbf{b}_i^\top\mathbf{\Sigma}_{yy}\mathbf{b}_j = \delta_{ij}, \qquad \mathbf{a}_i^\top\mathbf{\Sigma}_{xy}\mathbf{b}_j = \sigma_i\delta_{ij}, \qquad i, j = 1, \ldots, \min(n, m). \tag{43}$$

The first two of these relations are just the optimization constraints in multi-channel CCA (6). The last one can be proved by using the analytical solutions to CCA [15].

We expand $\mathbf{a}$ and $\mathbf{b}$ in CCA bases:

$$\mathbf{a} = \sum_{i=1}^{n}\alpha_i\mathbf{a}_i, \qquad \mathbf{b} = \sum_{i=1}^{m}\beta_i\mathbf{b}_i. \tag{44}$$

We can write the objective in terms of this expansion:

$$\begin{aligned} &\max_{\{\alpha_i,\beta_i\}} \sum_{i=1}^{\min(m,n)} \alpha_i\beta_i\mathbf{a}_i^\top\mathbf{\Sigma}_{xy}\mathbf{b}_i, \\ \text{s.t.}\ &\sum_i \alpha_i^2 = 1,\ \sum_i \beta_i^2 = 1, \\ &(\alpha_i + \beta_i)\left(1 + \mathbf{a}_i^\top\mathbf{\Sigma}_{xy}\mathbf{b}_i\right) = 0, \qquad i = 1, \ldots, d-1. \end{aligned} \tag{45}$$

Note that $\mathbf{a}_i^\top\mathbf{\Sigma}_{xy}\mathbf{b}_i \geq 0$ (if not we can flip the sign of one of $\mathbf{a}_i$ or $\mathbf{b}_i$ to get a higher value of the original CCA objective (6)). This observation combined with the constraints imply that $\alpha_i + \beta_i = 0$ for $i = 1, \ldots, d-1$, which further implies that $\alpha_i\beta_i \leq 0$ for $i = 1, \ldots, d-1$. Therefore, to avoid potential negative terms in the sum $\sum_{i=1}^{\min(m,n)}\alpha_i\beta_i\mathbf{a}_i^\top\mathbf{\Sigma}_{xy}\mathbf{b}_i$, the optimal solution requires $\alpha_i\beta_i = 0$ for $i = 1, \ldots, d-1$. Further, $\alpha_i = \beta_i = 0$ is optimal for $i = 1, \ldots, d-1$ because otherwise less weight would be given to the remaining nonnegative terms in the summation.

Next, we observe that by definition $\mathbf{a}_i^\top\mathbf{\Sigma}_{xy}\mathbf{b}_i > \mathbf{a}_j^\top\mathbf{\Sigma}_{xy}\mathbf{b}_j$ if $i < j$. Therefore, (45) is maximized by setting $\alpha_d = \beta_d = 1$, and the remaining coefficients $\alpha_i = \beta_i = 0$, $i \neq d$. This can be seen by using the Lagrange multiplier method on the remaining constraints. □
